# Supplementary figures and images for: The prognostic value of the early neutrophil-to-lymphocyte ratio for 28-day mortality in sepsis patients: A machine learning-based investigation of the MIMIC database
Source: PLoS One. 2026 Jun 2;21(6):e0348676. doi: 10.1371/journal.pone.0348676 (PMC13229304; doi:10.1371/journal.pone.0348676)

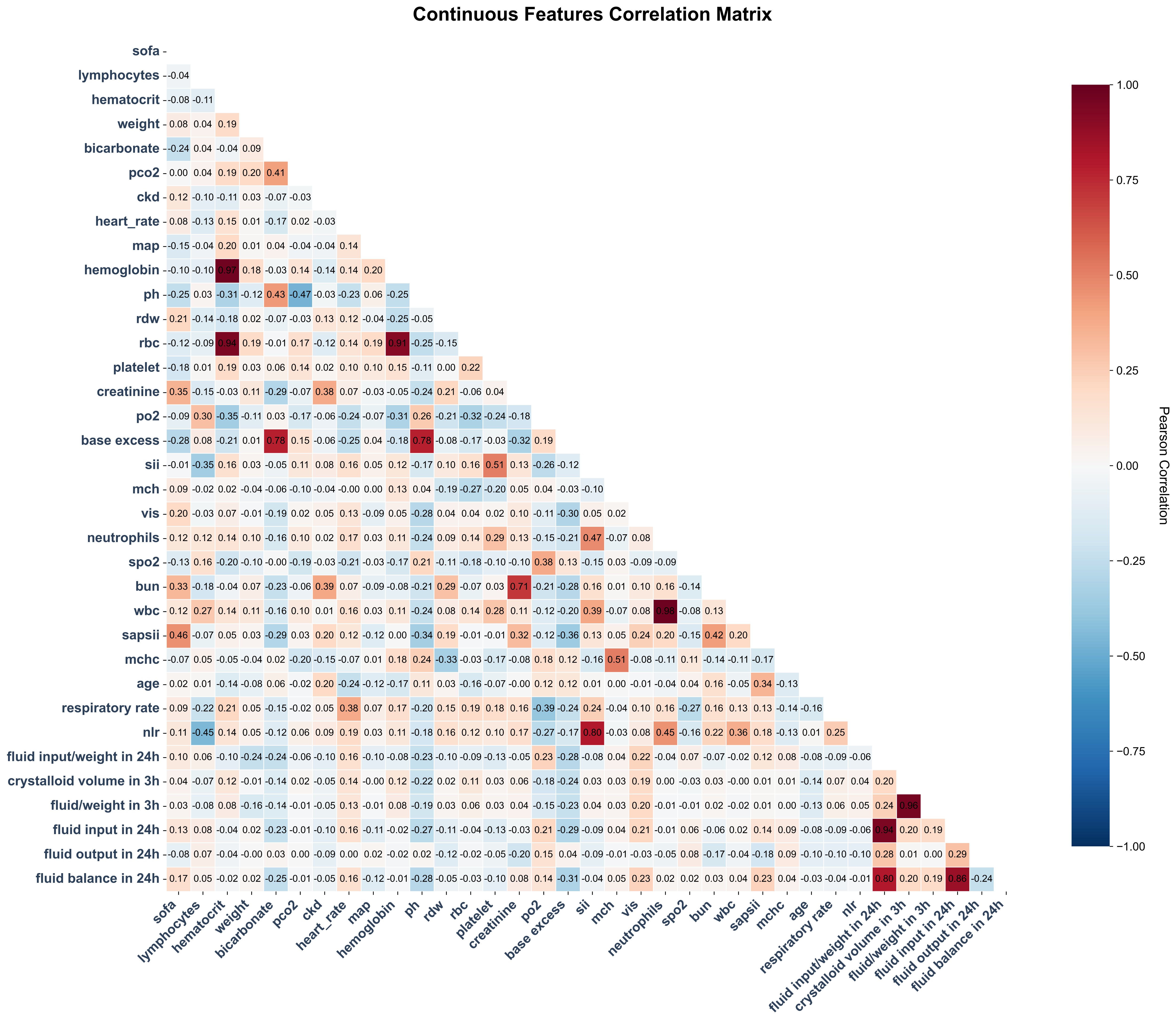

Supplement: S2 Fig — (TIF) [file pone.0348676.s002.tif]

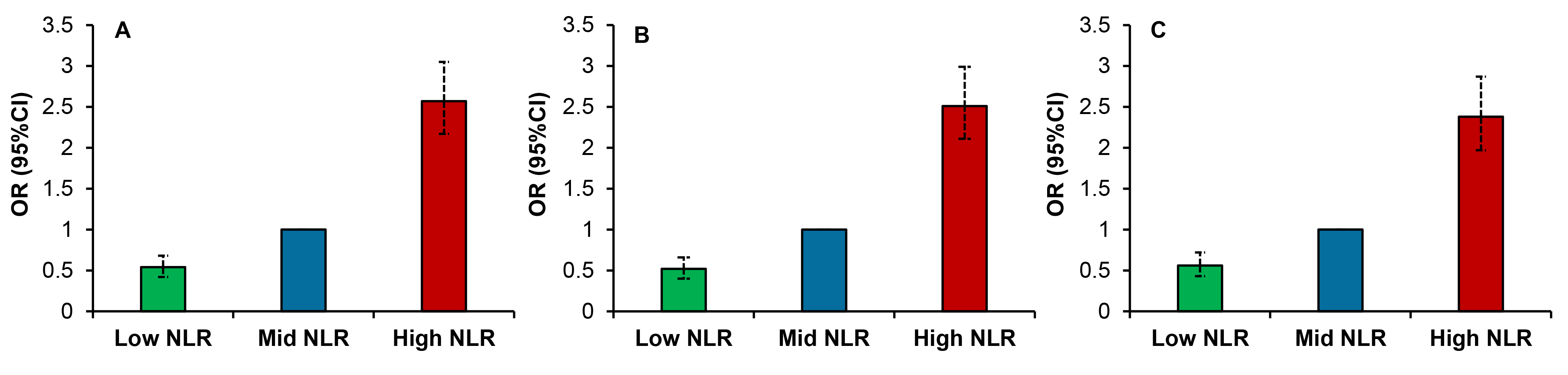

Supplement: S3 Fig — Panels A-C correspond to 28-day mortality (A), hospital mortality (B), and ICU mortality (C), respectively. Error bars represent 95% CIs, with the intermediate NLR concentration group serving as the reference category. (TIF) [file pone.0348676.s003.tif]

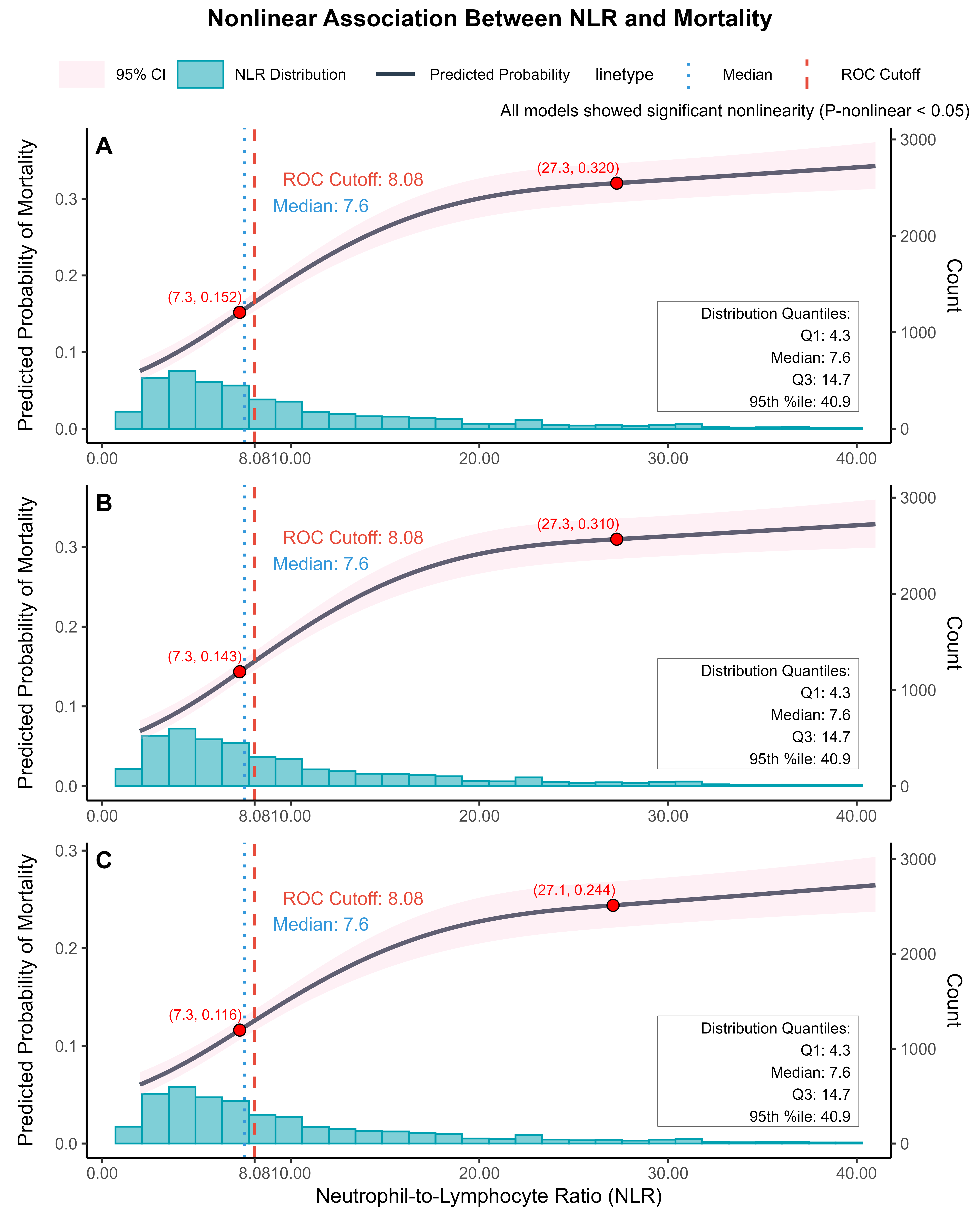

Supplement: S4 Fig — (A) 28-day mortality, (B) in-hospital mortality, and (C) ICU mortality. The solid line represents the adjusted odds ratio; the pink band indicates the 95% confidence interval. The histogram displays the distribution of the NLR values, with the median (7.6) and the ROC-derived optimal cutoff (8.08) marked. The nonlinear association was statistically significant for all of the endpoints (P for nonlinearity < 0.05). (TIFF) [file pone.0348676.s004.tiff]
