# Supplementary material for: The prognostic value of the early neutrophil-to-lymphocyte ratio for 28-day mortality in sepsis patients: A machine learning-based investigation of the MIMIC database
Source: PLoS One. 2026 Jun 2;21(6):e0348676. doi: 10.1371/journal.pone.0348676 (PMC13229304; doi:10.1371/journal.pone.0348676)
Supplement: S1 Table — (PDF) [file pone.0348676.s005.pdf]

S1 Table. Missing rate for demographics and clinical variables extracted from the database during the observation period.

Variable	Missing Ratio	Number of Missing
PaO2/FiO2 Ratio	70.45%	3083
Albumin	66.64%	2916
Lactate	51.33%	2246
SpO2	31.33%	1371
MAP	30.96%	1355
Heart Rate	30.62%	1340
PCO2	30.60%	1339
Base Excess	30.58%	1338
PO2	30.55%	1337
PH	30.55%	1337
Respiratory Rate	29.32%	1283
Bicarbonate	4.50%	197
Creatinine	4.41%	193
BUN	4.39%	192
RDW	0.30%	13
MCH	0.21%	9
MCHC	0.21%	9
Hemoglobin	0.18%	8
RBC	0.16%	7
Hematocrit	0.09%	4
Gender	0.00%	0
Age	0.00%	0
SOFA	0.00%	0
SOFA.Respiration	0.00%	0
SOFA.Coagulation	0.00%	0
SOFA.Hepatic	0.00%	0
SOFA.Cardiovascular	0.00%	0
SOFA.Neurologic	0.00%	0
SOFA.Kidney	0.00%	0
SAPSII	0.00%	0
Antibiotic Initiation	0.00%	0
LOS_ Hospital	0.00%	0
LOS_ICU	0.00%	0
Weight	0.00%	0
Hospital Mortality	0.00%	0
ICU Mortality	0.00%	0
28d Mortality	0.00%	0
WBC	0.00%	0
Platelet	0.00%	0
Lymphocytes	0.00%	0
Neutrophils	0.00%	0
SII	0.00%	0
NLR	0.00%	0
VIS	0.00%	0
Ventilation Status	0.00%	0
Duration of Ventilation	0.00%	0
Dialysis Type	0.00%	0
Crystalloid Volume in 3h	0.00%	0
Fluid/Weight in 3h	0.00%	0
Fluid Input in 24h	0.00%	0

Fluid Output in 24h	0.00%	0
Fluid Balance in 24h	0.00%	0
Fluid Input/Weight in 24h	0.00%	0
Diabetes	0.00%	0
Hypertension	0.00%	0
Heart Failure	0.00%	0
AMI	0.00%	0
COPD	0.00%	0
CKD	0.00%	0
Atrial Fibrillation	0.00%	0
Cerebral Infarction	0.00%	0
Cerebral Hemorrhage	0.00%	0
Thrombosis	0.00%	0
