# Supplementary material for: The prognostic value of the early neutrophil-to-lymphocyte ratio for 28-day mortality in sepsis patients: A machine learning-based investigation of the MIMIC database
Source: PLoS One. 2026 Jun 2;21(6):e0348676. doi: 10.1371/journal.pone.0348676 (PMC13229304; doi:10.1371/journal.pone.0348676)
Supplement: S2 Table — (PDF) [file pone.0348676.s006.pdf]

S7 Table. Comparison of Key Variables Before and After Imputation Using the misForest Algorithm.

Variable	Before imputation			After imputation		
	Missing (%)	Mean (SD)	Median (IQR)	Missing (%)	Mean (SD)	Median (IQR)
SpO2	31.33%	96.61 (4.40)	98.00 (95.00, 100.00)	0	97.15 (3.79)	98.43 (96.00, 99.39)
MAP	30.96%	82.72 (19.07)	81.00 (70.00, 93.00)	0	81.57 (16.15)	79.34 (73.10, 88.00)
Heart Rate	30.62%	93.18 (21.08)	91.00 (79.00, 107.00)	0	90.60 (18.47)	86.55 (79.00, 101.00)
PCO2	30.60%	42.32 (10.71)	41.00 (36.00, 46.00)	0	42.04 (9.21)	41.00 (37.48, 45.00)
Base Excess	30.58%	-1.67 (5.37)	0.00 (-4.00, 1.00)	0	-1.77 (4.77)	-1.00 (-4.00, 1.00)
PO2	30.55%	224.52 (129.87)	207.00 (101.50, 329.00)	0	207.18 (113.90)	175.60 (119.99, 285.00)
PH	30.55%	7.35 (0.10)	7.37 (7.30, 7.42)	0	7.36 (0.09)	7.37 (7.32, 7.41)
Respiratory Rate	29.32%	20.63 (6.46)	20.00 (16.00, 24.00)	0	19.62 (5.80)	18.00 (15.83, 22.62)
Bicarbonate	4.50%	21.89 (4.72)	22.00 (19.00, 24.00)	0	21.90 (4.65)	22.00 (19.00, 24.00)
Creatinine	4.41%	1.5 (1.53)	1.00 (0.70, 1.60)	0	1.49 (1.50)	1.00 (0.70, 1.50)
BUN	4.39%	26.51 (23.22)	19.00 (13.00, 31.00)	0	26.30 (22.84)	18.00 (13.00, 30.00)
RDW	0.30%	14.63 (2.20)	14.00 (13.20, 15.40)	0	14.63 (2.20)	14.00 (13.20, 15.40)
MCH	0.21%	30.23 (2.63)	30.30 (28.90, 31.70)	0	30.23 (2.63)	30.30 (28.90, 31.70)
MCHC	0.21%	32.99 (1.66)	33.10 (31.90, 34.10)	0	32.99 (1.66)	33.10 (31.90, 34.10)
Hemoglobin	0.18%	10.59 (2.37)	10.30 (8.88, 12.00)	0	10.59 (2.37)	10.30 (8.88, 12.00)
RBC	0.16%	3.52 (0.81)	3.43 (2.93, 4.03)	0	3.52 (0.81)	3.43 (2.93, 4.03)
Hematocrit	0.09%	32.11 (7.13)	31.40 (26.90, 36.40)	0	32.11 (7.13)	31.40 (26.90, 36.40)

Variables are sorted in descending order of original missingness. The MissForest algorithm was used for imputation. For variables with high initial missingness (>30%), the post-imputation medians and IQRs remained within clinically plausible ranges. IQR, interquartile range; SD, standard deviation.
