# Supplementary material for: The prognostic value of the early neutrophil-to-lymphocyte ratio for 28-day mortality in sepsis patients: A machine learning-based investigation of the MIMIC database
Source: PLoS One. 2026 Jun 2;21(6):e0348676. doi: 10.1371/journal.pone.0348676 (PMC13229304; doi:10.1371/journal.pone.0348676)
Supplement: S3 Table — (PDF) [file pone.0348676.s007.pdf]

S3 Table. Selected features and discarded features of collinearity assessment in XGBoost modeling

Selected features	Discarded features	Pearson correlation coefficient
WBC	Neutrophils	0.98
Hematocrit	Hemoglobin	0.97
Fluid/Weight in 3h	Crystalloid Volume in 3h	0.96
Hematocrit	RBC	0.94
Fluid Input in 24h	Fluid Input/Weight in 24h	0.94
Hemoglobin	RBC	0.91
Fluid Balance in 24h	Fluid Input in 24h	0.86
NLR	SII	0.80
Fluid Balance in 24h	Fluid Input/Weight in 24h	0.80
Base excess	bicarbonate	0.78
Base excess	pH	0.78
BUN	Creatinine	0.71
