# Supplementary material for: The prognostic value of the early neutrophil-to-lymphocyte ratio for 28-day mortality in sepsis patients: A machine learning-based investigation of the MIMIC database
Source: PLoS One. 2026 Jun 2;21(6):e0348676. doi: 10.1371/journal.pone.0348676 (PMC13229304; doi:10.1371/journal.pone.0348676)
Supplement: S4 Table — (PDF) [file pone.0348676.s008.pdf]

**S4 Table. Performance evaluation of XGBoost model across training and test datasets**

| Performance Metrics | Value |
|---|---|
| **Training Set** | |
| ROC_AUC | 0.93 |
| PR_AUC | 0.74 |
| Accuracy | 0.86 |
| Sensitivity | 0.81 |
| Specificity | 0.87 |
| F1-score | 0.68 |
| **Test Set** | |
| ROC_AUC | 0.88 |
| PR_AUC | 0.60 |
| Accuracy | 0.82 |
| Sensitivity | 0.70 |
| Specificity | 0.84 |
| F1-score | 0.58 |
