# Supplementary material for: The prognostic value of the early neutrophil-to-lymphocyte ratio for 28-day mortality in sepsis patients: A machine learning-based investigation of the MIMIC database
Source: PLoS One. 2026 Jun 2;21(6):e0348676. doi: 10.1371/journal.pone.0348676 (PMC13229304; doi:10.1371/journal.pone.0348676)
Supplement: S5 Table — (PDF) [file pone.0348676.s009.pdf]

**S5 Table. Feature importances in the XGBoost model.**

| Feature | Importance |
|---|---|
| SAPSII | 59.15 |
| Fluid Output in 24h | 30.52 |
| PO2 | 26.41 |
| Respiratory Rate | 26.29 |
| Dialysis Type_CRRT | 25.01 |
| VIS | 23.12 |
| Creatinine | 21.06 |
| NLR | 20.11 |
| RDW | 19.04 |
| Lymphocytes | 15.19 |
| Hematocrit | 14.13 |
| MAP | 13.66 |
| Cerebral Infarction_Yes | 13.62 |
| Base Excess | 13.19 |
| Weight | 12.95 |
| Heart Rate | 12.94 |
| Age | 12.86 |
| Fluid Balance in 24h | 12.84 |
| MCH | 10.79 |
| SpO2 | 10.31 |
| MCHC | 9.75 |
| PCO2 | 9.50 |
| Fluid/Weight in 3h | 9.31 |
| Dialysis Type_No | 8.97 |
| Platelet | 8.68 |
| SOFA | 8.39 |
| WBC | 8.32 |
| Atrial Fibrillation_Yes | 8.30 |
| CKD | 7.52 |
