# Supplementary material for: The prognostic value of the early neutrophil-to-lymphocyte ratio for 28-day mortality in sepsis patients: A machine learning-based investigation of the MIMIC database
Source: PLoS One. 2026 Jun 2;21(6):e0348676. doi: 10.1371/journal.pone.0348676 (PMC13229304; doi:10.1371/journal.pone.0348676)
Supplement: S6 Table — (PDF) [file pone.0348676.s010.pdf]

S6 Table. Subgroup analysis for the association of NLR with 28-day mortality, hospital mortality and ICU mortality.

X = NLR Std.		28-day mortality			Hospital mortality			ICU mortality		
Subgroup	N	OR (95%CI)	P-value	P-interaction	OR (95%CI)	P-value	P-interaction	OR (95%CI)	P-value	P-interaction
**Age**										
18-44	677	0.99 (0.75-1.28)	0.940	Ref.	0.96 (0.72-1.25)	0.785	Ref.	0.98 (0.72-1.30)	0.877	Ref.
45-64	1747	1.18 (1.01-1.36)	0.032	0.221	1.20 (1.03-1.39)	0.019	0.171	1.12 (0.95-1.31)	0.156	0.377
> = 65	1952	1.17 (1.03-1.33)	0.019	0.127	1.11 (0.98-1.27)	0.102	0.114	1.16 (1.02-1.32)	0.029	0.164
**VIS**										
0	2785	1.17 (1.03-1.34)	0.021	Ref.	1.17 (1.01-1.33)	0.034	Ref.	1.16 (0.98-1.34)	0.078	Ref.
0-10	557	1.16 (0.94-1.48)	0.174	0.802	1.10 (0.88-1.37)	0.386	0.452	1.08 (0.84-1.33)	0.509	0.430
> 10	1034	1.12 (0.98-1.28)	0.096	0.083	1.11 (0.97-1.28)	0.138	0.044	1.13 (0.99-1.30)	0.080	0.086
**Gender**										
Female	1616	1.14 (0.99-1.31)	0.061	Ref.	1.10 (0.96-1.27)	0.178	Ref.	1.13 (0.99-1.30)	0.080	Ref.
Male	2760	1.18 (1.05-1.32)	0.006	0.738	1.16 (1.03-1.31)	0.013	0.585	1.14 (1.01-1.30)	0.042	0.957
**Cerebral infarction**										
No	3916	1.15 (1.05-1.27)	0.004	Ref.	1.10 (1.00-1.21)	0.059	Ref.	1.11 (1.00-1.23)	0.041	Ref.
Yes	460	1.15 (0.91-1.45)	0.227	0.802	1.30 (1.04-1.65)	0.024	0.268	1.31 (1.02-1.68)	0.039	0.654
**Atrial fibrillation**										
No	3127	1.16 (1.04-1.30)	0.010	Ref.	1.15 (1.02-1.29)	0.021	Ref.	1.16 (1.03-1.31)	0.017	Ref.
Yes	1249	1.13 (0.97-1.31)	0.122	0.488	1.07 (0.92-1.25)	0.378	0.612	1.07 (0.91-1.25)	0.418	0.853
**SAPSII**										
< = 29	1104	1.37 (1.02-1.80)	0.039	Ref.	1.31 (0.98-1.73)	0.071	Ref.	1.27 (0.86-1.78)	0.213	Ref.
>29	3272	1.15 (1.05-1.26)	0.002	0.181	1.12 (1.03-1.23)	0.012	0.147	1.14 (1.04-1.25)	0.005	0.264
**SOFA**										
< = 4	3042	1.25 (1.12-1.40)	< 0.001	Ref.	1.21 (1.08-1.36)	0.001	Ref.	1.24 (1.10-1.40)	<0.001	Ref.
5-8	1136	1.01 (0.87-1.18)	0.853	0.080	1.00 (0.86-1.17)	0.958	0.101	0.99 (0.84-1.16)	0.929	0.053
> = 9	198	1.23 (0.79-2.02)	0.369	0.733	1.24 (0.79-2.05)	0.370	0.452	1.04 (0.65-1.76)	0.887	0.886
**FO in 24h**										
< 1475	1090	1.07 (0.94-1.21)	0.320	Ref.	1.02 (0.90-1.17)	0.733	Ref.	1.03 (0.90-1.18)	0.687	Ref.
1475-3420	2194	1.26 (1.09-1.46)	0.001	0.010	1.21 (1.05-1.40)	0.010	0.013	1.22 (1.05-1.42)	0.010	0.013
> 3420	1092	1.19 (0.89-1.53)	0.225	0.155	1.27 (0.96-1.64)	0.090	0.101	1.33 (1.00-1.72)	0.051	0.053
**Creatinine**										
<1.5	3191	1.13 (1.00-1.27)	0.052	Ref.	1.10 (0.97-1.24)	0.121	Ref.	1.18 (1.05-1.34)	0.008	Ref.
1.5-3.0	764	1.25 (1.04-1.50)	0.016	0.547	1.18 (0.98-1.43)	0.078	0.383	1.13 (0.93-1.36)	0.217	0.128

>3.0	421	1.21 (0.99-1.49)	0.063	0.529	1.22 (0.99-1.50)	0.060	0.612	1.07 (0.86-1.34)	0.524	0.162
**Lymphocytes**										
< 0.75	1082	1.10 (0.99-1.21)	0.069	Ref.	1.09 (0.98-1.20)	0.114	Ref.	1.08 (0.97-1.20)	0.166	Ref.
0.75-1.93	2200	1.15 (0.87-1.52)	0.317	0.703	1.22 (0.92-1.62)	0.174	0.452	1.20 (0.89-1.62)	0.225	0.506
> 1.93	1094	1.88 (0.55-6.22)	0.307	0.086	2.91 (0.83-10.13)	0.096	0.030	1.91 (0.52-6.77)	0.323	0.086
**Dialysis type**										
No	3866	1.15 (1.04-1.28)	0.007	Ref.	1.11 (1.00-1.23)	0.055	Ref.	1.15 (1.03-1.28)	0.014	Ref.
Yes	510	1.20 (1.01-1.44)	0.035	0.199	1.23 (1.03-1.49)	0.022	0.383	1.13 (0.95-1.35)	0.174	0.103
**FB in 24h**										
< = 2493.27	2369	1.21 (1.08-1.36)	<0.001	Ref.	1.20 (1.07-1.35)	0.002	Ref.	1.21 (1.07-1.36)	0.002	Ref.
>2493.27	2007	1.08 (0.93-1.24)	0.311	0.199	1.02 (0.87-1.18)	0.834	0.106	1.02 (0.87-1.19)	0.829	0.103
**MCH**										
<27	403	1.25 (0.99-1.59)	0.064	Ref.	1.11 (0.86-1.42)	0.422	Ref.	1.13 (0.86-1.49)	0.391	Ref.
>=27	3973	1.13 (1.02-1.24)	0.014	0.221	1.12 (1.02-1.24)	0.021	0.620	1.12 (1.01-1.24)	0.029	0.603
**RDW**										
< = 14.63	2796	1.15 (1.00-1.32)	0.057	Ref.	1.19 (1.04-1.37)	0.014	Ref.	1.23 (1.07-1.42)	0.005	Ref.
> 14.63	1580	1.22 (1.08-1.37)	0.001	0.620	1.12 (1.00-1.27)	0.055	0.756	1.09 (0.96-1.23)	0.171	0.516
**Hematocrit**										
< = 32.11	2399	1.19 (1.03-1.36)	0.014	Ref.	1.10 (0.96-1.27)	0.167	Ref.	1.06 (0.92-1.23)	0.435	Ref.
> 32.11	1977	1.17 (1.03-1.32)	0.014	0.181	1.17 (1.04-1.33)	0.013	0.483	1.22 (1.07-1.38)	0.002	0.955
**BE**										
<-6	694	1.03 (0.87-1.21)	0.739	Ref.	0.97 (0.82-1.15)	0.769	Ref.	0.96 (0.80-1.14)	0.629	Ref.
-6--2	1061	1.09 (0.92-1.28)	0.307	0.083	1.14 (0.96-1.34)	0.146	0.044	1.13 (0.94-1.34)	0.202	0.057
>-2	2621	1.38 (1.19-1.60)	<0.001	<0.001	1.27 (1.09-1.48)	0.002	<0.001	1.27 (1.08-1.49)	0.003	<0.001
**PO2**										
<=60	115	1.67 (0.78-3.61)	0.190	Ref.	1.83 (0.87-3.93)	0.108	Ref.	1.39 (0.72-2.82)	0.338	Ref.
60-300	3288	1.15 (1.05-1.26)	0.003	0.270	1.12 (1.02-1.23)	0.018	0.176	1.12 (1.02-1.23)	0.021	0.264
>300	973	1.04 (0.62-1.59)	0.854	0.774	0.96 (0.56-1.47)	0.858	0.452	1.06 (0.62-1.62)	0.817	0.853
**Heart rate**										
<60	112	4.93 (1.85-17.14)	<0.001	Ref.	6.59 (2.03-34.42)	<0.001	Ref.	6.92 (2.15-33.01)	<0.001	Ref.
60-100	3143	1.22 (1.08-1.37)	0.001	0.127	1.16 (1.03-1.31)	0.017	0.055	1.15 (1.01-1.30)	0.038	0.057
>100	1121	1.06 (0.93-1.21)	0.401	0.037	1.07 (0.94-1.23)	0.296	0.019	1.09 (0.95-1.25)	0.214	0.025
**MAP**										
<65	427	1.14 (0.89-1.45)	0.303	Ref.	1.09 (0.85-1.39)	0.496	Ref.	1.17 (0.90-1.49)	0.237	Ref.
65-110	3720	1.22 (1.10-1.35)	<0.001	0.216	1.18 (1.06-1.30)	0.002	0.208	1.17 (1.05-1.30)	0.005	0.506

>110	229	0.82 (0.54-1.18)	0.286	0.181	0.95 (0.63-1.39)	0.794	0.398	0.93 (0.61-1.36)	0.724	0.264
**Respiratory rate**										
<12	118	1.41 (0.58-3.65)	0.430	Ref.	1.49 (0.57-8.55)	0.425	Ref.	1.60 (0.59-5.03)	0.340	Ref.
12-20	2720	1.30 (1.12-1.50)	<0.001	0.157	1.26 (1.09-1.47)	0.002	0.044	1.31 (1.12-1.53)	<0.001	0.071
>20	1538	1.08 (0.96-1.20)	0.199	0.037	1.05 (0.94-1.17)	0.402	0.008	1.04 (0.92-1.17)	0.503	0.013
**Weight**										
< = 88.81	2411	1.16 (1.04-1.31)	0.011	Ref.	1.12 (0.99-1.26)	0.072	Ref.	1.15 (1.02-1.30)	0.025	Ref.
> 88.81	1965	1.18 (1.02-1.35)	0.022	0.802	1.18 (1.02-1.36)	0.022	0.894	1.14 (0.98-1.32)	0.085	0.884
