# Supplementary material for: The prognostic value of the early neutrophil-to-lymphocyte ratio for 28-day mortality in sepsis patients: A machine learning-based investigation of the MIMIC database
Source: PLoS One. 2026 Jun 2;21(6):e0348676. doi: 10.1371/journal.pone.0348676 (PMC13229304; doi:10.1371/journal.pone.0348676)
Supplement: S7 Table — (PDF) [file pone.0348676.s011.pdf]

**S7 Table. Abbreviations**

| Abbreviations | Full name |
|---|---|
| SOFA | Sequential Organ Failure Assessment |
| SAPSII | Simplified Acute Physiology Score II |
| MAP | Mean Arterial Pressure |
| SpO2 | Peripheral Oxygen Saturation |
| WBC | White Blood Cell |
| NLR | Neutrophil-to-Lymphocyte Ratio |
| SII | Systemic Immune-Inflammation Index |
| PO2 | Partial Pressure of Oxygen |
| PCO2 | Partial Pressure of Carbon Dioxide |
| BE | Base Excess |
| BUN | Blood Urea Nitrogen |
| RBC | Red Blood Cell |
| RDW | Red Cell Distribution Width |
| MCH | Mean Corpuscular Hemoglobin |
| MCHC | Mean Corpuscular Hemoglobin Concentration |
| AMI | Acute Myocardial Infarction |
| COPD | Chronic Obstructive Pulmonary Disease |
| CKD | Chronic Kidney Disease |
| CRRT | Continuous Renal Replacement Therapy |
| VIS | Vasoactive-Inotropic Score |
| LOS of Hospital | Length of Stay in Hospital |
| LOS of ICU | Length of Stay in Intensive Care Unit |
| FO in 24h | Fluid Output within 24h |
| FB in 24h | Fluid Balance within 24h |
| ROC | Receiver Operating Characteristic |
| PR | Precision-Recall |
| AUC | Area Under the PR Curve |
