# Supplementary material for: The prognostic value of the early neutrophil-to-lymphocyte ratio for 28-day mortality in sepsis patients: A machine learning-based investigation of the MIMIC database
Source: PLoS One. 2026 Jun 2;21(6):e0348676. doi: 10.1371/journal.pone.0348676 (PMC13229304; doi:10.1371/journal.pone.0348676)
Supplement: Attachment — Submitted filename: Response to Reviewers.docx [file pone.0348676.s013.docx]

**Response to Reviewer 1-Comment 1**

**Reviewer’s Comment:**

*This is a well-conducted retrospective study that leverages a large public database and advanced machine learning techniques to investigate the prognostic value of the neutrophil-to-lymphocyte ratio (NLR) in sepsis. The manuscript is generally well-written, and the methodological approach, including the two-stage feature selection process using Boruta and XGBoost, is a strength. However, several important issues need to be addressed to strengthen the manuscript's claims and contextualize its findings within the existing literature. Firstly, the novelty statement should be moderated. While the combination of methods is robust, claiming this as the "first large-scale retrospective study employing machine learning-based feature selection to evaluate the ability of the NLR" may be too absolute. Recent research has increasingly applied ML techniques to similar prognostic questions in sepsis using the MIMIC database. For instance, a 2024 study in Scientific Reports used the MIMIC-IV database to explore the triglyceride-glucose (TyG) index, another accessible biomarker, and its association with mortality in septic patients, employing similar analytical techniques including restricted cubic splines and subgroup analysis (DOI: 10.1038/s41598-024-75050-8). Reframing the contribution to highlight the specific, rigorous feature selection pipeline and the detailed exploration of NLR's biphasic predictive pattern across clinical subgroups would provide a more accurate and compelling narrative.*

**Author’s Response:**

We sincerely thank the reviewer for the thoughtful and constructive feedback. We fully agree that the original wording of our novelty statement was overly absolute and insufficiently contextualized within the growing body of machine learning (ML) research using the MIMIC database. We are grateful for the reviewer’s suggestion to cite the relevant 2024 Scientific Reports study on the TyG index (DOI: 10.1038/s41598-024-75050-8), which indeed represents a methodologically similar and high-quality contribution. We have now revised the opening paragraph of the Discussion section to:

1.**Remove all absolute claims** (e.g., “To our knowledge, this is the first…”) and instead position our work within the broader context of recent ML-based prognostic studies in sepsis.

2.**Explicitly cite and acknowledge** the TyG index study, using it as an example of how ML is increasingly employed to reevaluate accessible biomarkers-thereby clarifying that our contribution lies not in being the first to use such techniques, but in applying them specifically to the NLR with a particularly rigorous feature selection protocol and a novel focus on clinical phenotype stratification.

3.Reframe the core contribution around two specific, defensible innovations:

a) **Methodological:** a robust two-stage (Boruta + XGBoost) feature selection pipeline combined with SHAP-based marginal contribution analysis.
b) **Clinical:** the first detailed description of a biphasic, phenotype-specific predictive pattern of the NLR, which distinguishes inflammation-driven mortality risk (evident in patients with preserved organ function) from organ failure-driven mortality (where NLR loses its predictive utility).

We believe this revised framing more accurately reflects the study’s genuine contributions and appropriately acknowledges prior work in the field. We are grateful to the reviewer for guiding us toward a more precise and compelling narrative.

**Response to Reviewer 1-Comment 2**

**Reviewer’s Comment:**

Several methodological aspects require further clarification. The decision to use only the first NLR value within six hours of ICU admission is pragmatic but represents a significant limitation, as a single static measurement may not capture the dynamic inflammatory state critical in sepsis. The discussion should more thoroughly acknowledge that this "early" snapshot might miss peak values or trends that carry greater prognostic weight. Furthermore, while the use of the missForest package for imputation is appropriate, the manuscript would benefit from a brief validation statement or supplementary table comparing the distribution of key variables before and after imputation to assure readers of the integrity of the imputed dataset. The model performance is commendable, but the discussion would be enriched by a more direct comparison with other contemporary biomarker-based models developed on similar cohorts. For example, studies have examined composite indices like the creatinine-to-albumin ratio (CAR) for sepsis-associated acute kidney injury, finding non-linear associations with mortality, which echoes your findings on NLR's biphasic pattern (DOI: 10.3389/fcimb.2025.1602921). Situating your XGBoost model's performance relative to these emerging indicators would better define its relative clinical utility.

**Author’s Response:**

We sincerely thank the reviewer for these thoughtful and constructive methodological suggestions. We have carefully addressed each of the three specific concerns: **(1) limitation of single NLR measurement, (2) validation of missForest imputation, and (3) contextualization against other emerging biomarker-based models, as detailed below.** All corresponding revisions have been clearly marked in the tracked-change version of the manuscript.

**1. Limitation of a Single Early NLR Measurement**

**Reviewer’s concern:**

The use of only the first NLR value within six hours of ICU admission is a pragmatic but potentially significant limitation, as a static measurement may not reflect the dynamic inflammatory trajectory of sepsis.

**Our response:**

We fully agree with the reviewer. Although we had briefly acknowledged this limitation in the original manuscript, we have now substantially expanded this discussion in the Limitations section. Specifically, we now explicitly state that a single baseline measurement captures only a static snapshot of the inflammatory state and may underestimate the prognostic value of NLR trajectories or peak values, which could better reflect the evolving host response. We also explicitly call for future studies incorporating serial NLR measurements or longitudinal trajectory modeling to further validate and refine our findings. This revision directly aligns with our overall conclusion that the clinical utility of NLR lies in dynamic monitoring rather than isolated static assessment.

**Action taken:**

a) **Manuscript location:** *Discussion – Limitations* (please see track changed)

**2.Validation of missForest Imputation**

**Reviewer’s concern:**

While the use of missForest for imputation is appropriate, the manuscript would benefit from a brief validation statement or supplementary table comparing the distribution of key variables before and after imputation to assure readers of the integrity of the imputed dataset.

**Our response:**

We appreciate this suggestion for enhanced transparency. In response, we performed a systematic comparison of the distributions of key continuous and categorical variables before and after imputation using the missForest algorithm. The results are now presented in S2 Table.

a) For variables with **low missingness (<5%)**, the distributions remained virtually identical.
b) For variables with **higher missing rates (approximately 30%)**, such as SpO₂, MAP, heart rate, and blood gas parameters, we observed a modest convergence in dispersion (SD, IQR)—an expected behavior of model‑based imputation when recovering missing values from observed patterns.
c) **Critically, all post‑imputation means, medians, and quartiles remained within clinically plausible and physiologically coherent ranges**, and no systematic shift would alter the clinical interpretation of these parameters (e.g., median SpO₂: 98.0% → 98.4%; median MAP: 81.0 mmHg → 79.3 mmHg).

We have added a brief validation statement in the Methods section (Data extraction) and direct readers to S2 Table for full details.

**Action taken:**

a) **Manuscript location:** *Methods – Data extraction* (please see track changed).
b) **Supplementary** **material:** Added S2 Table (“Comparison of Key Variables Before and After Imputation Using the MissForest Algorithm”).

**3. Comparison with Other Emerging Biomarker-Based Models**

**Reviewer’s concern:**

The discussion would be enriched by a more direct comparison with other contemporary biomarker-based models developed on similar cohorts—particularly studies examining the creatinine-to-albumin ratio (CAR) in sepsis-associated acute kidney injury, which reported non‑linear associations with mortality echoing our biphasic NLR pattern. Situating our XGBoost model’s performance relative to these emerging indicators would better define its relative clinical utility.

**Our response:**

We thank the reviewer for guiding us to contextualize our findings within the broader landscape of novel prognostic biomarkers. We have now substantially expanded the Discussion to include a dedicated paragraph that directly compares:

- **Study populations and sample sizes** (our N = 4,376 vs. 1,257–2,712 in comparator studies);
- **Model performance** (our XGBoost ROC‑AUC = 0.875, PR‑AUC = 0.603 vs. CAR‑based AUCs of 0.68-0.75; TyG studies did not report AUC but reported hazard ratios of 1.4-1.8);
- **Non‑linear patterns** (our biphasic NLR‑mortality curve with inflection points at NLR = 7.3 and 27.3 vs. the J‑shaped CAR‑mortality curve with inflection at CAR = 1.2 mg/dL, and the non‑linear TyG‑mortality curve reported by Lou et al. [29] with inflection at TyG = 8.9);
- **Practical advantages of NLR:** zero additional cost, routine availability from complete blood count, and direct biological linkage to innate‑adaptive immune imbalance-a pathophysiological axis distinct from insulin resistance (TyG) or renal‑inflammatory crosstalk (CAR).

We emphasize that these biomarkers are not mutually exclusive but rather capture complementary aspects of sepsis pathophysiology, and their combined use may enable more refined phenotype‑specific risk stratification. Our model’s superior discriminative performance underscores the value of integrating a simple, inexpensive marker like NLR within a robust machine learning framework.

**Action taken:**

- **Manuscript location:** *Discussion – second paragraph* (please see track changed).

We are confident that these revisions have substantially improved the methodological transparency, scholarly depth, and clinical contextualization of our manuscript. We thank the reviewer again for these insightful suggestions, which we believe have greatly strengthened our work.

**Response to Reviewer 1-Comment 3**

**Reviewer’s Comment:**

The most substantial limitation is the lack of external validation, which is rightly mentioned but deserves greater emphasis in the discussion. The reliance solely on the MIMIC database, while common, introduces risks related to selection bias and protocol homogeneity from a single healthcare system. The conclusions about NLR's predictive value, particularly its diminished utility in severe organ failure, must be framed as hypothesis-generating and require validation in independent, prospective cohorts. Additionally, please ensure all figures and tables are correctly numbered and embedded in the main text for review, and standardize the formatting of abbreviations throughout (e.g., SAPS II vs. SAPSII). In the results, when stating survivors had better vital signs, please specify which parameters showed significant differences. The statistical methods section should explicitly state which p-values were adjusted using the Benjamini-Hochberg procedure.

**Author’s Response:**

We thank the reviewer for these thoughtful and constructive comments. We have carefully addressed each point as follows:

1. **External validation and hypothesis‑generating framing.**

We fully agree that the absence of external validation is the most substantial limitation of our study. While we had acknowledged this in the original manuscript, the reviewer correctly notes that the emphasis was insufficient. In response, we have substantially strengthened the Limitations section to explicitly state that:

- Our single‑center MIMIC‑derived findings are subject to selection bias and protocol homogeneity;
- The proposed biphasic, phenotype‑dependent predictive pattern of the NLR must be regarded as hypothesis‑generating, not definitive;
- Independent validation in prospective, multi‑center cohorts is urgently required before any clinical implementation can be considered;
- Future external validation should specifically test the generalizability of the identified inflection points (NLR = 7.3 and 27.3) and the differential predictive utility across SOFA strata observed in our cohort.

We have also revised the Conclusions section to echo this cautious framing, explicitly stating that our conclusions remain hypothesis‑generating pending external validation. All changes are clearly marked in the tracked‑change manuscript.

2. **Figures and tables numbering/embedding.**

We have verified that all figures and tables are correctly numbered and that each is explicitly cited in the main text.

3. **Abbreviation standardization.**

We have standardized all abbreviations throughout the manuscript. Specifically, “SAPSII” has been uniformly corrected to “SAPS II” in all instances. Other abbreviations (SOFA, CRRT, VIS, etc.) were already consistent and remain unchanged.

4. **Specification of vital signs in Results.**

We have revised the Results – Baseline characteristics section to explicitly list which vital signs showed significant differences and which did not.

5. **Clarification of Benjamini‑Hochberg adjustment.**

We have now explicitly clarified the application of the Benjamini–Hochberg procedure in the Statistical analysis section. Specifically, we state that:

a) For multiple comparisons across categorical covariates in baseline tables (e.g., comorbidities, organ dysfunction components, treatment categories), p‑values were adjusted using the Benjamini–Hochberg method to control the false discovery rate.
b) For interaction p‑values derived from subgroup analyses, the same FDR correction was applied to comprehensively evaluate potential effect modifiers.

**Response to Reviewer 1-Comment 4**

**Reviewer’s Comment:**

The discussion effectively interprets the biphasic nature of the NLR's predictive value. To further strengthen it, consider integrating the concept that the predictive weight of inflammatory markers like NLR may be context-dependent, overshadowed by organ dysfunction in advanced disease. This resonates with research on other composite markers that combine inflammatory and nutritional/renal dimensions, such as the ratio of neutrophil counts to the prognostic nutritional index (NPNR), which has shown J- or V-shaped associations with mortality in septic patients, highlighting the complex interplay between inflammation and host reserve (DOI: 10.3389/fcimb.2025.1603104; DOI: 10.3389/fnut.2025.1602016). Citing such work would help build a more nuanced theoretical framework for why NLR's utility plateaus in critical illness. Finally, the reference list should be formatted consistently according to the journal's style, and including some of the more recent, relevant studies suggested here would update the scholarly context.

**Author’s Response:**

We thank the reviewer for this thoughtful and constructive suggestion. We fully agree that the context‑dependent nature of inflammatory biomarkers is a critical concept that deserves explicit articulation within our theoretical framework. In response, we have:

**1.Expanded the Discussion to incorporate the NPNR analogy.**

We have added a new paragraph in the section of Discussion that explicitly links our findings on the NLR’s biphasic pattern to recent research on the neutrophil count‑to‑prognostic nutritional index ratio (NPNR). Both NLR and NPNR have been shown to exhibit J‑ or V‑shaped associations with mortality, suggesting a shared underlying principle: inflammatory markers are most informative when the host still possesses physiological reserve, and their predictive utility wanes once organ failure becomes the dominant driver of outcome. This convergence across distinct biomarker constructs strengthens the notion that risk stratification in sepsis must be context‑aware, accounting for the dynamic interplay between inflammation, nutrition, and organ function.

**2. Updated the reference list.**

We have added all suggested studies to the reference list and carefully reformatted all references to ensure strict adherence to the PLOS ONE style guidelines.

We believe these additions enrich the theoretical depth of our discussion and appropriately position our findings within a broader, emerging literature on composite inflammatory‑nutritional biomarkers in sepsis. We thank the reviewer for guiding us toward this more nuanced framework.

All changes are clearly marked in the tracked‑change version of the manuscript.
